# Human breast microbiome correlates with prognostic features and immunological signatures in breast cancer

**DOI:** 10.1186/s13073-021-00874-2

**Published:** 2021-04-16

**Authors:** Alice Tzeng, Naseer Sangwan, Margaret Jia, Chin-Chih Liu, Karen S. Keslar, Erinn Downs-Kelly, Robert L. Fairchild, Zahraa Al-Hilli, Stephen R. Grobmyer, Charis Eng

**Affiliations:** 1 Genomic Medicine Institute, Lerner Research Institute, Cleveland Clinic, Cleveland, OH 44195 USA; 2 Cleveland Clinic Lerner College of Medicine of Case Western Reserve University, Cleveland, OH 44195 USA; 3 Microbiome Composition and Analytics Core, Cleveland Clinic, Lerner Research Institute, Cleveland, OH 44195 USA; 4 Department of Inflammation and Immunity, Cleveland Clinic, Lerner Research Institute, Cleveland, OH 44195 USA; 5 Department of Anatomic Pathology, Cleveland Clinic, Robert J. Tomsich Pathology and Laboratory Medicine Institute, Cleveland, OH 44195 USA; 6 Department of General Surgery, Cleveland Clinic, Cleveland, OH 44195 USA; 7 Cleveland Clinic Abu Dhabi, Oncology Institute, Abu Dhabi, United Arab Emirates; 8 Cleveland Clinic, Taussig Cancer Institute, Cleveland, OH 44195 USA; 9 Department of Genetics and Genome Sciences, Case Western Reserve University School of Medicine, Cleveland, OH 44106 USA; 10 Germline High Risk Focus Group, Case Comprehensive Cancer Center, Case Western Reserve University School of Medicine, Cleveland, OH 44106 USA

**Keywords:** Host microbial interactions, Biomarkers, Microbiota, Mammary carcinoma, Tumor microenvironment

## Abstract

**Background:**

Currently, over half of breast cancer cases are unrelated to known risk factors, highlighting the importance of discovering other cancer-promoting factors. Since crosstalk between gut microbes and host immunity contributes to many diseases, we hypothesized that similar interactions could occur between the recently described breast microbiome and local immune responses to influence breast cancer pathogenesis.

**Methods:**

Using 16S rRNA gene sequencing, we characterized the microbiome of human breast tissue in a total of 221 patients with breast cancer, 18 individuals predisposed to breast cancer, and 69 controls. We performed bioinformatic analyses using a DADA2-based pipeline and applied linear models with White’s *t* or Kruskal–Wallis *H*-tests with Benjamini–Hochberg multiple testing correction to identify taxonomic groups associated with prognostic clinicopathologic features. We then used network analysis based on Spearman coefficients to correlate specific bacterial taxa with immunological data from NanoString gene expression and 65-plex cytokine assays.

**Results:**

Multiple bacterial genera exhibited significant differences in relative abundance when stratifying by breast tissue type (tumor, tumor adjacent normal, high-risk, healthy control), cancer stage, grade, histologic subtype, receptor status, lymphovascular invasion, or node-positive status, even after adjusting for confounding variables. Microbiome–immune networks within the breast tended to be bacteria-centric, with sparse structure in tumors and more interconnected structure in benign tissues. Notably, *Anaerococcus*, *Caulobacter*, and *Streptococcus*, which were major bacterial hubs in benign tissue networks, were absent from cancer-associated tissue networks. In addition, *Propionibacterium* and *Staphylococcus*, which were depleted in tumors, showed negative associations with oncogenic immune features; *Streptococcus* and *Propionibacterium* also correlated positively with T-cell activation-related genes.

**Conclusions:**

This study, the largest to date comparing healthy versus cancer-associated breast microbiomes using fresh-frozen surgical specimens and immune correlates, provides insight into microbial profiles that correspond with prognostic clinicopathologic features in breast cancer. It additionally presents evidence for local microbial–immune interplay in breast cancer that merits further investigation and has preventative, diagnostic, and therapeutic potential.

**Supplementary Information:**

The online version contains supplementary material available at 10.1186/s13073-021-00874-2.

## Background

Despite the prevalence of breast cancer, many cases are unrelated to known risk factors. Furthermore, not all individuals with genetic predisposition or exposure to documented environmental factors develop disease [1], indicating an urgent need to identify additional determinants of breast carcinogenesis. Increasing evidence suggests that the gut microbiome plays a significant role in immunity and other essential host processes and that microbial perturbation (dysbiosis) contributes to disease states, including malignancy [2]. Recently, tissue-resident microbes at other sites such as the skin, oral cavity, and respiratory tracts have been discovered [3]. While several groups, including ours, have found that the breast harbors a diverse microbiome that differs significantly in patients with and without breast cancer [4–9], the function of microbiota at extra-intestinal tissues remains poorly understood.

Immune homeostasis in the gut relies on constant crosstalk between the microbiota and host immune cells. By influencing metabolism, inflammation, and immune responses, the microbiome can regulate cancer initiation and progression at both local and distant sites [10]. Intriguingly, recent work using mouse models demonstrated that gut microbial composition can determine susceptibility to mammary carcinoma [11]. In humans, a large case–control study showed that increasing cumulative antibiotic use corresponded with increased breast cancer risk [12]. With major efforts focused on examining regional and systemic effects of the gut microbiome, much less consideration has been given to putative interactions between extra-intestinal microbial populations and local immunity, especially as they relate to carcinogenesis. Though bacterial 16S rRNA and lipopolysaccharides can associate with immune cells in breast tumors, their impact on immune function remains unclear [13]. Conceivably, the breast microbiota may influence breast cancer development and growth not only by modulating local estrogen levels [14], but also by shaping inflammatory responses and immune trafficking in the tumor microenvironment.

To better understand the role of regional microbiome–immune system interplay in breast tumorigenesis, we compared the breast microbiota and immune signatures of patients with breast cancer to those of healthy individuals, focusing on microbial and immunological differences that co-varied with one another and with clinicopathologic factors. We thereby sought to address the hypothesis that patients with versus without breast cancer have distinct microbial and immunological profiles and associations, which highlight potential connections between the breast microbiome and local cancer-related immune responses.

## Methods

### Patient enrollment and tissue collection

Fresh-frozen breast tissues were requested from three tissue biorepositories (Cleveland Clinic Breast Center Microbiome Biorepository, Cooperative Human Tissue Network, and Case Comprehensive Cancer Center Human Tissue Procurement Facility). Specimens were obtained using standard biorepository protocols from female patients undergoing surgery for breast cancer, reduction mammoplasty, or prophylactic mammoplasty who provided written informed consent. If available, breast cancer (tumor) and adjacent normal breast tissue (tumor adjacent normal) pairs from the same donor were included. Tissue from patients without breast cancer (healthy control) was verified by a pathologist to be free of malignant cells. Patients with genetic predisposition (pathogenic gene carrier or first-degree relative with breast cancer) or past personal history of breast cancer were considered at high risk for breast cancer. Histopathological data were compiled from pathology reports. For patients recruited at the Cleveland Clinic, additional clinical history and information regarding breast cancer risk factors were acquired through a combination of patient interview and standardized questionnaire. Breast cancer staging was standardized using American Joint Committee on Cancer/Union for International Cancer Control 8th edition TNM pathologic stage criteria. Specimens were flash frozen and stored at − 80 °C until further processing. This study was approved by the Cleveland Clinic institutional review board (IRB #14-774 and 17-791).

To control for possible environmental microbial contamination, a specimen container filled with 5 ml of sterile saline or water was left open in the operating room during breast surgery at each institution from which specimens were collected (Cleveland Clinic, Hospital of the University of Pennsylvania, The Ohio State University Wexner Medical Center, University Hospitals Cleveland Medical Center, University of Virginia Medical Center). These environmental controls were also stored at − 80 °C and processed in parallel with tissue specimens.

### RNA extraction

Total RNA was extracted from frozen breast tissue using the RNeasy Lipid Tissue Mini Kit (Qiagen, Hilden, Germany) with the following modification. Samples were homogenized in 2-ml tubes with Lysing Matrix A (MP Biomedicals, Solon, OH, USA) and 1 ml QIAzol Lysis Reagent using a FastPrep-24 5G instrument (MP Biomedicals) with 3 runs of 30 s at 6 m/s. Subsequently, RNA preparation was performed according to the manufacturer’s protocol, including the optional on-column DNase digestion step. RNA concentrations and quality (A260/280, A260/230) were determined by spectrophotometry (Thermo Scientific NanoDrop 1000, Waltham, MA, USA), and samples were stored at − 80 °C until further analysis.

### NanoString gene expression analysis

Total RNA was hybridized to the Human Immunology v2 Panel CodeSet and processed on an nCounter GEN2 Digital Analyzer (NanoString Technologies, Seattle, WA, USA) per manufacturer’s instructions. Normalization, background subtraction, and hybridization/binding intensity correction were performed using nCounter Advanced Analysis 2.0 software (NanoString Technologies), and resulting values were log_2_-transformed for downstream analysis. K-means clustering of normalized gene expression based on one minus the Pearson correlation was performed using Morpheus (Broad Institute, Cambridge, MA, USA). Differential pathway analysis, KEGG pathway overlay, and immune cell type profiling were also conducted using Advanced Analysis software. False discovery rates (FDR) for differential gene expression were adjusted using the Benjamini–Yekutieli method.

### Multiplex cytokine assay

Frozen breast tissue was placed into chilled 2-ml homogenization tubes containing 3.0 mm zirconium beads (Benchmark Scientific, Edison, NJ, USA). Cold PBS with 2X complete protease inhibitor cocktail (Roche, Basel, Switzerland) was added in a ratio of 4 μl/mg (tumor) or 3 μl/mg (non-tumor) tissue, and samples were homogenized using a FastPrep-24 5G instrument (MP Biomedicals) with 3 runs of 30 s at 6 m/s. After sequential centrifugation (14,000×*g* for 15 min at 4 °C, transfer to a new tube, then 14,000×*g* for 10 min at 4 °C), supernatant aliquots were taken for protein quantification using a BCA assay kit (Thermo Scientific) in accordance with the manufacturer’s protocol. PBS with protease inhibitor was added to normalize the protein concentrations of all supernatant samples to 1.5 mg/ml, and samples were stored at − 80 °C until analysis. Cytokine expression was evaluated using the Human Cytokine/Chemokine Array 65-Plex Assay performed by Eve Technologies (Calgary, AB, Canada). Background fluorescence was subtracted from all samples, and data were log_10_-transformed prior to further analysis.

### DNA extraction

Bacterial genomic DNA was isolated from frozen breast tissue and environmental controls using the QIAamp PowerFecal Pro DNA Kit (Qiagen) with minor modifications as follows. To minimize contamination from environmental microbial DNA, all pipettes, pipette tips, and non-enzymatic kit components were UV-irradiated for at least 1 h prior to use, and extraction was performed in a dedicated laminar flow hood (AirClean 600 PCR Workstation, Creedmoor, NC, USA) [15]. For homogenization, samples were placed into 2-ml tubes with Lysing Matrix A (MP Biomedicals) along with 800 μl Solution CD1 and processed with 4 runs of 45 s at 6 m/s in a FastPrep-24 5G instrument (MP Biomedicals). The rest of the protocol proceeded per the manufacturer’s instructions. Buffer-only negative controls and extraction positive controls (ZymoBIOMICS Microbial Community Standard; Zymo Research, Irvine, CA, USA) were processed identically in parallel. DNA concentrations and quality (A260/280, A260/230) were determined by spectrophotometry (Thermo Scientific NanoDrop 1000), and samples were stored at − 20 °C until 16S rRNA gene library preparation.

### 16S rRNA gene sequencing

Bacterial 16S rRNA gene V3-V4 and V7-V9 regions were PCR-amplified using the QIAseq 16S Region Panel (Qiagen) according to kit instructions for amplification of samples with low bacterial content followed by PCR cleanup. Pipettes and plasticware were UV-irradiated before use, and PCR reactions were set up in a dedicated laminar flow hood. For negative controls, nuclease-free water was substituted for the DNA template. The QIAseq 16S/ITS 96-Index Kit (Qiagen) was used to complete library construction. After cleanup (Ampure XP beads; Beckman Coulter, Brea, CA, USA) and quantification (Qubit dsDNA broad-range assay; Thermo Scientific), libraries were normalized to 2 nM, pooled, denatured, and diluted to a final concentration of 10 pM. Libraries were validated on a Bioanalyzer DNA 1000 chip (Agilent Technologies, Santa Clara, CA, USA) and sequenced with an Illumina MiSeq (San Diego, CA, USA) using the V3 reagent kit (2 × 300 bp paired-end) at the Case Western Reserve University Genomics Core.

### Bioinformatic analysis

Demultiplexed fastq files were processed with a Divisive Amplicon Denoising Algorithm (DADA) 2-based pipeline [16] as follows. After dereplication was conducted, the output, a feature table containing amplicon sequence variants (ASVs) and associated abundances, was generated based on forward reads. Chimeric and shifted sequences were removed through DADA2, and ASVs present in the environmental and negative controls were subtracted from all samples as previously described [17, 18] using R (Additional file 1: Figure S1). For this purpose, ASVs have an advantage over traditional operating taxonomic units (OTUs) because while OTU-based approaches consolidate similar sequences into consensus units, the ASV approach treats exact sequences as unique units, thereby enabling the removal of contaminating sequences without significantly affecting relevant reads [16]. A total of 11,000 ASVs were present in environmental and negative controls; however, > 99% of these ASVs were assigned to the domain Eukaryota by the DADA2 taxonomy classifier and were thus excluded in downstream analyses. Sequences were then classified against Silva [19]. After removing eukaryotic sequences and trimming ASVs with < 3 total reads, α-diversity indices within group categories were calculated using *phyloseq* [20] and plotted using *ggpubr* (https://rpkgs.datanovia.com/ggpubr/) in R. The *metagenomeSeq* R package was used to apply cumulative sum scaling normalization followed by linear modeling to identify differentially abundant taxa across groups after correcting for specimen collection site (hospital), age, and race [21].

Using the *psych* R package [22], microbiome–immune networks were constructed based on pairwise Spearman correlations between genus-level bacterial relative abundances and either NanoString gene expression or multiplex cytokine assay data. To enrich for putative biologically relevant associations, only taxa detected in at least 2 samples were included, and analyses focused on immune data that were significantly different in tumor versus healthy control tissue (FDR <  0.05). Furthermore, a filter was applied to select only associations with Spearman coefficient |*r*| > 0.2 and *p* <  0.05. Networks were visualized using a default force-directed layout algorithm in *igraph* [23].

### 16S rRNA gene quantitative PCR

Total bacterial load was measured by qPCR using the following universal 16S rRNA gene primers: Uni340F (ACTCCTACGGGAGGCAGCAGT) and Uni514R (ATTACCGCGGCTGCTGGC) [24, 25]. Each 50-μl PCR reaction contained 4 μl of DNA template or environmental/negative control, 25 μl of QuantiTect SYBR Green Master Mix (Qiagen), 1.5 μl each of 10 μM forward and reverse primers, and 18 μl of nuclease-free water. The following thermal cycling program was performed on an Applied Biosystems 7500 Real-Time PCR System (Foster City, CA, USA): initial 15-min denaturation step at 95 °C; 40 cycles at 94 °C for 15 s, 60 °C for 30 s, and 72 °C for 30 s. The PCR product size was 197 bp, and product purity was verified by melting curve analysis. Absolute quantification of bacterial DNA was then performed using standard curves constructed with *Escherichia coli* reference genomic DNA (ATCC, Manassas, VA, USA). Although qPCR measures 16S rRNA gene copies per sample instead of actual bacterial numbers or colony-forming units, these values are directly related, showing considerable correlation [24, 26]. All standards and controls were run in duplicate; all samples were run in triplicate.

### Immunohistochemistry and image analysis

For a random subset of cases from the Cleveland Clinic (19 breast cancer cases; 6 prophylactic mastectomy cases; 5 reduction mammoplasty cases), corresponding archival formalin-fixed, paraffin-embedded tissue was sectioned at 5 μm. The immunohistochemistry double stain was completed using a DISCOVERY ULTRA automated stainer (Roche). In brief, antigen retrieval was performed using a tris/borate/EDTA buffer (DISCOVERY CC1; Roche #950-500), pH 8.0 to 8.5, for 64 min at 95 °C. Slides were incubated with a 1:100 dilution of anti-FOXP3 antibody (clone 236A/E7; Abcam, Cambridge, UK) for 40 min at 37 °C. FOXP3 was visualized using the OmniMap anti-mouse HRP secondary antibody (Roche #760-4310) and the ChromoMap DAB detection kit (Roche #760-159). Slides were then double-labeled with a pre-diluted anti-CD8 antibody solution (clone SP57; Roche #790-4460) for 40 min at 37 °C. CD8 was visualized using UltraMap anti-rabbit AP (Roche #760-4314) and the DISCOVERY Red AP detection kit (Roche #760-228). Lastly, the slides were counterstained with hematoxylin and bluing.

Immunostained slides were scanned with an Aperio AT2 automated slide scanner (Leica Biosystems, Wetzlar, Germany). The resulting SVS image files were viewed and manually annotated by a pathologist using Aperio ImageScope software 12.3.3 (Leica Biosystems) to delineate tumor regions. Subsequently, images were analyzed using CaloPix software (TRIBVN Healthcare, Paris, France) with the Tissue Recognition 4.0.0 and Cell Recognition 4.1.0 macros as follows. Using a selection of pathologist-annotated images, the machine-learning software was trained to create a decision model based on color, texture, and edge criteria on the color-deconvolved channels DAB and eosin that assigned a probability of belonging to the “tumor,” “stroma,” or “background” classes to each image pixel. Each pixel was then classified with the label scoring the highest probability. Similarly, a machine-learning decision model based on color, texture, and edge criteria was created to assign a probability of belonging to the “cell” class to each image pixel. The maxima of the resulting probability map generated a point centered on each cell, and detected cells were classified into CD8^+^ or FOXP3^+^ cells using the point neighborhood on the color-deconvolved channels DAB and eosin. Cell densities were quantified in the normal breast and intratumoral compartments using CaloPix as previously described [27].

### Statistical analysis

Data were analyzed using GraphPad Prism 8.4.3 (San Diego, CA, USA) and R v4.0.2. Statistical tests were based on two-sided comparisons with significance set at *p* <  0.05 unless otherwise specified. A sample size of ≥ 200 patients with breast cancer was chosen to enable the assessment of up to 20 clinical variables using multivariable regression by the rule of 10 [28]. To compare patient characteristics, the Kruskal–Wallis test was used for continuous data and Fisher’s exact test for categorical data. Pairwise bacterial α-diversity comparisons were made using one-way ANOVA. White’s *t* or Kruskal–Wallis *H*-tests with Benjamini–Hochberg FDR correction for multiple comparisons were conducted to identify differentially abundant bacterial taxa when stratifying by clinical variables. When evaluating differential abundance based on tumor-specific variables, FDR-corrected values < 0.1 were considered significant in order to avoid overlooking important taxa with variable presence [13, 29, 30]. Comparisons of total bacterial load and of immune cell densities were performed using the Mann–Whitney test or Kruskal–Wallis test with posthoc Dunn test. Cytokine multiplex assay data were analyzed using two-way ANOVA with posthoc Tukey test. Finally, Spearman rank-order correlation was used to evaluate associations between genus-level bacterial relative abundances and immune cell type scores (estimated abundances).

## Results

### Patient characteristics

Our study included fresh-frozen breast specimens from 221 patients with breast cancer and 87 patients without breast cancer. Of the patients without breast cancer, 18 were categorized as being at high risk for breast cancer based on genetic predisposition or patient history; specimens from these patients were nonetheless verified as histologically free of malignant cells by a pathologist. Patient demographic and clinicopathologic characteristics are shown in Table 1. Since significant differences in age and race were present among patient groups, these variables, along with specimen collection site (hospital), were treated as confounders (covariates) in subsequent statistical analyses. For the 66 patients recruited at the Cleveland Clinic, additional information on known breast cancer risk factors and oral antibiotic use was available, which showed no significant differences among the patient groups aside from breast cancer family history (Table 2).

**Table 1:** Patient characteristics

Variable	Cancer (*n* = 221)	High-risk (*n* = 18)	Healthy control (*n* = 69)	***p***-value
**Age at surgery (years)**	57 (47–66)	45 (36–51)	38 (26–47)	< 0.0001
**Race**^**a**^				< 0.0001
Caucasian	191 (86%)	14 (78%)	37 (54%)	
African American	26 (12%)	4 (22%)	27 (40%)	
Others	4 (2%)		4 (6%)	
**TNM stage**^**a**^				
0	3 (1.5%)			
1	124 (63%)			
2	44 (22%)			
3	27 (13.5%)			
**Grade**^**a**^				
1	24 (11%)			
2	86 (40%)			
3	107 (49%)			
**Histology**				
IDC	164 (74%)			
ILC	27 (12%)			
IDC + ILC	12 (6%)			
Others	18 (8%)			
**ER**^**+a**^	164 (82%)			
**PR**^**+a**^	143 (72%)			
**HER2**^**+a**^	15 (8%)			
**TNBC**^**a**^	30 (15%)			
**LVI**^**a**^	90 (43%)			
**Node positive**^**a**^	109 (52%)			

Data are presented as number of patients (%) or median (interquartile range)

^a^Missing data: age (*n* = 1), race (*n* = 1), stage (*n* = 23), grade (*n* = 4), ER (*n* = 21), PR (*n* = 22), HER2 (*n* = 37), TNBC (*n* = 23), LVI (*n* = 14), and node-positive status (*n* = 10). Percentages are calculated from the total number of patients with known values

*IDC*, invasive ductal carcinoma; *ILC*, invasive lobular carcinoma; *ER*, estrogen receptor; *PR*, progesterone receptor; *HER2*, human epidermal growth factor 2; *TNBC*, triple-negative breast cancer; *LVI*, lymphovascular invasion

**Table 2:** Breast cancer risk factors and antibiotic use in patients recruited at the Cleveland Clinic

Variable	Cancer (*n* = 48)	High-risk (*n* = 7)	Healthy control (*n* = 11)	***p***-value
**Age at menarche (years)**	13 (12–13)	12 (12–13)	11 (10–12)	0.230
**Postmenopausal**	28 (58%)	5 (71%)	4 (36%)	0.367
**Gravity/parity**	2 (1–3.8)/2 (1–3)	2 (1–3)/2 (0–3)	1.5 (0–2.5)/1 (0–2.3)	0.309/0.397
**OCP or HRT use**^**a**^	33 (69%)	6 (100%)	4 (57%)	0.248
**Family history of breast cancer**^**a**^	26 (54%)	4 (67%)	0 (0%)	0.001
**Alcohol use**^**a**^				0.421
Frequent	19 (40%)	2 (28.5%)	4 (40%)	
Occasional	12 (25%)	2 (28.5%)	5 (50%)	
None	17 (35%)	3 (43%)	1 (10%)	
**Last oral antibiotic use**^**a**^				0.985
< 1 month ago	6 (17%)	1 (17%)	2 (20%)	
1–6 months ago	12 (34%)	2 (33%)	4 (40%)	
> 6 months ago	17 (49%)	3 (50%)	4 (40%)	

Data are presented as number of patients (%) or median (interquartile range)

^a^Missing data: OCP or HRT use (*n* = 5), family history (*n* = 1), alcohol use (*n* = 1), and antibiotic use (*n* = 15). Percentages are calculated from the total number of patients with known values

*OCP*, oral contraceptive pill; *HRT*, hormone replacement therapy

### Breast tumor tissue exhibits distinct microbiome composition

Since the breast tissue microbiome has a relatively low biomass, we took extensive precautions to minimize the potential impact of bacterial contaminants as described in the “Methods” section, including collecting and analyzing multiple environmental and negative controls alongside the tissue samples [15]. Furthermore, amplicon sequence variants detected in the environmental and negative controls were computationally removed from subsequent analyses (Additional file 1: Figure S1). Absolute quantification of total bacterial load using qPCR with universal 16S rRNA gene primers revealed significantly greater bacterial abundance in all breast tissue types versus environmental and negative controls (Additional file 1: Figure S2a). Moreover, the total bacterial load was similar across tissue types (Additional file 1: Figure S2b) and, as expected, was several orders of magnitude lower in comparison to that found in high-biomass sites such as the gut and stool [25, 31, 32].

**Fig. 1:**
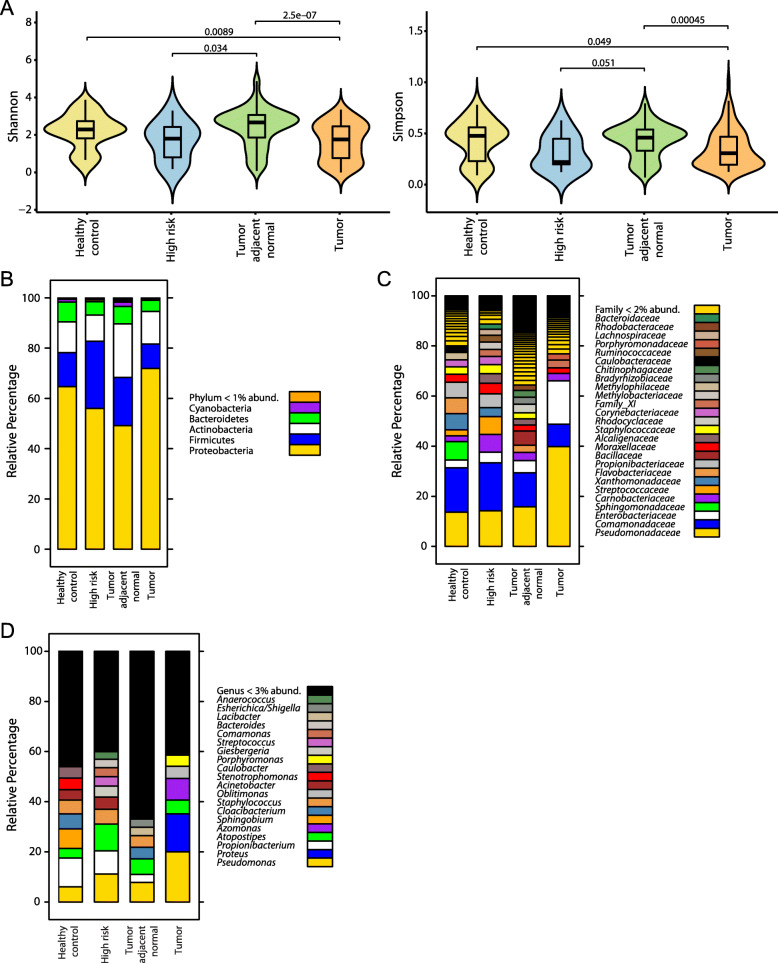
Breast bacterial community composition varies by patient breast cancer status and tissue type. **a** Bacterial α-diversity as measured by Shannon and Simpson diversity indices within breast tissue from patients with (tumor, tumor adjacent normal) versus without (healthy control, high-risk) breast cancer. Violin plots show median and interquartile range. *p-*values result from one-way ANOVA tests. Taxonomic composition of the breast microbiome, depicted as average relative abundances at the phylum (**b**), family (**c**), and genus (**d**) levels for each tissue type

To quantify overall differences in breast microbial diversity between tissue types, we applied two measures of α-diversity, defined as within-group taxonomic richness and evenness, and found that tumor tissue exhibited significantly decreased α-diversity compared to tumor adjacent normal or healthy control tissue (Fig. 1a). Interestingly, high-risk tissue also demonstrated a trend toward reduced α-diversity, particularly in comparison to tumor adjacent normal tissue. We next sought to characterize breast microbial differences at various taxonomic levels. In all tissue types, the top bacterial phylum was Proteobacteria, with either Firmicutes or Actinobacteria being the next most abundant (Fig. 1b). Differences between tissue types were more apparent at lower taxonomic levels: for example, compared to other tissues, tumor tissue contained a much higher percentage of the families *Pseudomonadaceae* and *Enterobacteriaceae* (Fig. 1c). At the genus level, *Pseudomonas* constituted a greater proportion of the breast microbiome in tumor versus other tissues, and *Proteus*, the second most abundant genus in tumor tissue, was largely absent from non-tumor tissues (Fig. 1d). *Porphyromonas* and *Azomonas* also had a higher abundance in tumor compared with other tissues. Conversely, *Propionibacterium* and *Staphylococcus* were prominent constituents of healthy control, high-risk, and tumor adjacent normal tissues but were scarce in tumor tissue. Healthy control tissue was further characterized by the marked presence of *Stenotrophomonas* and *Caulobacter*, genera that were not detected above the 3% abundance threshold in other tissues.

**Fig. 2:**
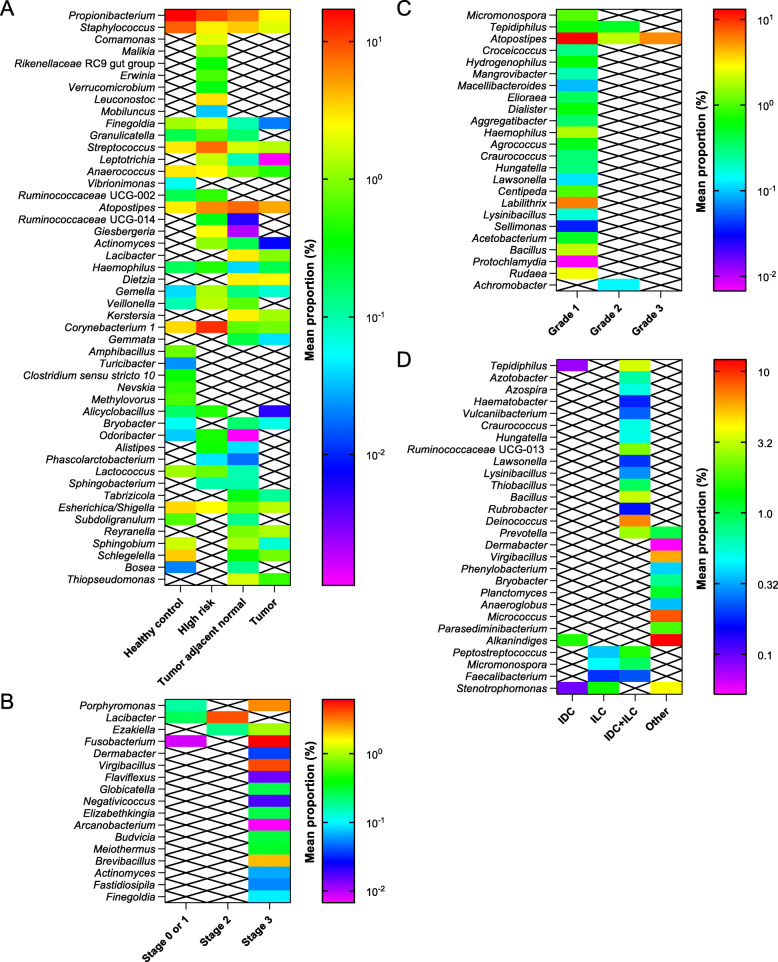
Specific bacterial genera correlate with clinicopathologic features. Mean relative abundances (proportions) of bacterial genera that were differentially present in distinct breast tissue types (**a**) and in breast tumors stratified by cancer stage (**b**), histologic grade (**c**), and histologic subtype (**d**). Stages 0 and 1 were combined for analysis due to the very small number of samples classified as stage 0. Crossed-out boxes indicate samples for which specific genera were not detected. Color bars vary on a logarithmic scale. All genera shown had FDR-corrected *p*-value < 0.05 by Kruskal–Wallis *H*-test after adjustments for age, race, and hospital

Subsequently, we identified 48 bacterial genera that were differentially abundant after stratifying by tissue type and adjusting for known confounders and microbiome-influencing factors (patient age, race, hospital) [9, 33, 34]. Benign tissue samples (healthy control, high-risk) displayed similar microbiome composition and were characterized by greater mean relative abundances of 11 genera (*Propionibacterium*, *Finegoldia*, *Granulicatella*, *Streptococcus*, *Anaerococcus*, *Ruminococcaceae* UCG-002, *Corynebacterium* 1, *Alicyclobacillus*, *Odoribacter*, *Lactococcus*, *Esherichica*/*Shigella*) compared to cancer-associated samples (tumor, tumor adjacent normal) (Fig. 2a, Additional file 2: Table S1). Nonetheless, there were subtle differences between the microbial profiles of healthy control and high-risk tissues, including certain genera that were present in healthy control yet absent from high-risk samples (e.g., *Vibrionimonas*, *Amphibacillus*) and vice versa. Of the genera present in both types of cancer-associated tissue, the majority (17/22) were enriched in tumor adjacent normal versus tumor tissue, emphasizing that the microbiome of breast tumors is distinct not only from that of healthy control tissue, but also from that of adjacent normal tissue.

### Multiple bacterial genera are significantly associated with prognostic breast tumor features

While previous studies have described associations between specific breast microbial taxa and prognostic breast cancer features such as stage [9], histologic grade [8], receptor status [4, 7, 9, 13, 35], and lymphovascular invasion [4], none has examined these associations together in the same study. Our comparatively large sample size enabled us to use multivariate regression to identify bacterial genera with statistically different abundances when stratifying by cancer stage, grade, histologic subtype, receptor status, lymphovascular invasion, or lymph node status and adjusting for patient age, race, and hospital. In particular, *Porphyromonas*, *Lacibacter*, *Ezakiella*, and *Fusobacterium* were more abundant in higher versus lower stage tumors, and 13 genera were present only in stage 3 tumors (Fig. 2b, Additional file 2: Table S1). Similarly, multiple genera were significantly associated with histologic grade, with a number of genera present only in grade 1 tumors (Fig. 2c, Additional file 2: Table S1). Distinct microbial profiles also correlated with each histologic tumor subtype: for instance, invasive ductal carcinoma (IDC) was characterized by the presence of *Tepidiphilus*, *Alkanindiges*, and *Stenotrophomonas*, while invasive lobular carcinoma (ILC) samples contained *Peptostreptococcus*, *Micromonospora*, *Faecalibacterium*, and *Stenotrophomonas* (Fig. 2d, Additional file 2: Table S1).

**Fig. 3:**
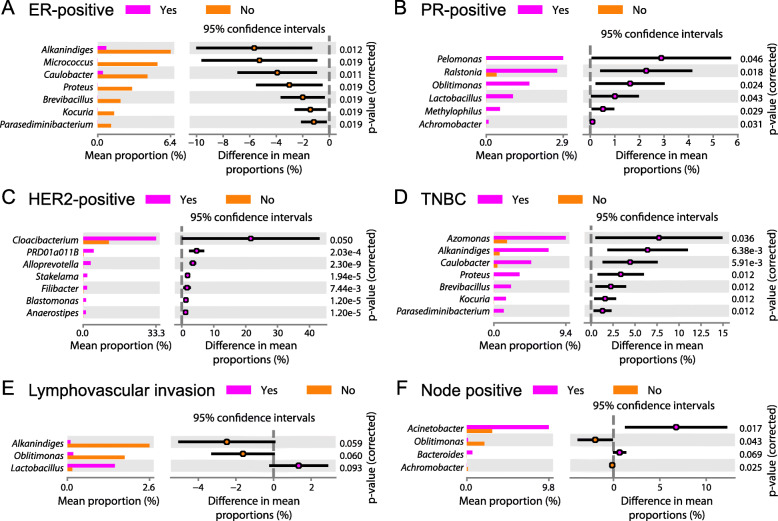
Specific bacterial genera correlate with breast tumor receptor status and metastatic potential. Mean relative abundances (proportions) of bacterial genera that were differentially present in ER positive versus negative (**a**), PR positive versus negative (**b**), HER2 positive versus negative (**c**), and TNBC versus non-TNBC (**d**) breast tumors, and in breast tumors with versus without lymphovascular invasion (**e**) and with versus without positive lymph nodes (**f**). All genera shown had FDR-corrected *p*-value < 0.1 by White’s *t* test after adjustments for age, race, and hospital

Upon stratifying samples by tumor receptor status, we noted that estrogen receptor (ER)-positive tumors consistently had lower abundances of 7 genera (*Alkanindiges*, *Micrococcus*, *Caulobacter*, *Proteus*, *Brevibacillus*, *Kocuria*, *Parasediminibacterium*) compared to ER-negative tumors (Fig. 3a). In contrast, 6 genera (*Pelomonas*, *Ralstonia*, *Oblitimonas*, *Lactobacillus*, *Methylophilus*, *Achromobacter*) showed associations with progesterone receptor (PR)-positive status (Fig. 3b). Human epidermal growth factor 2 (HER2)-positive tumors had significantly higher abundances of 7 genera (*Cloacibacterium*, *PRD01a011B*, *Alloprevotella*, *Stakelama*, *Filibacter*, *Blastomonas*, *Anaerostipes*) compared to HER2-negative tumors (Fig. 3c). Meanwhile, 6 of 7 genera that were relatively decreased in ER-positive tumors, along with the genus *Azomonas*, were enriched in triple-negative breast cancer (TNBC) tumors (Fig. 3d). We also identified taxonomic associations with markers of tumor metastatic potential. Lymphovascular invasion and node-positive status correlated with reduced abundance of *Oblitimonas* (Fig. 3e, f). Lymphovascular invasion further associated positively with *Lactobacillus* and negatively with *Alkanindiges*, while node-positive status associated positively with *Acinetobacter* and *Bacteroides* and negatively with *Achromobacter*. Collectively, these findings of shared as well as distinct bacterial profiles associated with prognostic breast tumor features suggest that breast microbiome–tumor interactions are complex and likely involve multiple factors.

### Breast microbial and immunological signatures co-vary with each other and form association networks

**Fig. 4:**
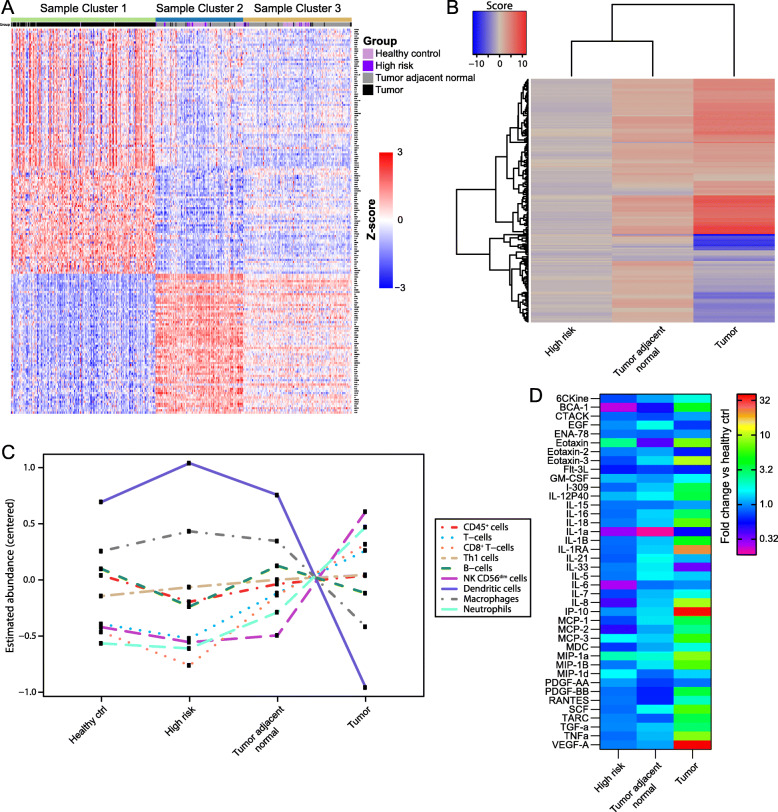
Breast tumor tissue exhibits a distinct immunological signature. **a** K-means clustering (*k* = 3) of 443 breast tissue samples by expression levels of immune-related genes as measured by NanoString. Genes with the greatest differential expression between tumor and healthy controls are shown (|fold change| > 2 and FDR < 0.05; *n* = 179 genes). Rows represent individual genes (log_2_ count normalized to standard deviations from the mean), and columns represent individual tissue samples. Cluster 1 is strongly enriched for tumor tissue. **b** Heatmap of directed global significance scores based on NanoString data showing 164 cellular pathways whose genes were overexpressed (red) or underexpressed (blue) in the indicated tissue type relative to healthy control tissue. **c** Estimated abundance of immune cell subsets in each tissue type based on stably expressed, specific marker genes present in the NanoString CodeSet. Abundance estimates are reported as the average log_2_ counts of marker genes for each cell subset that has been centered to have mean value 0; each unit increase corresponds to a doubling in abundance. **d** Cytokines present at significantly different levels in the indicated tissue types relative to healthy control tissue as measured by Milliplex assay (*p* < 0.05 by 2-way ANOVA with posthoc Tukey test; *n* = 40 cytokines). Color bar varies on a logarithmic scale

Given the gut microbiome’s well-defined role in shaping host immunity [10], we hypothesized that differential composition of the breast microbiome may influence the intratumoral immune microenvironment. To delineate the immunological landscape in our samples, we measured the expression of 579 immune-related genes in 443 breast tissue samples (196 tumor, 175 tumor adjacent normal, 17 high-risk, 55 healthy control) using NanoString. We applied an unsupervised learning algorithm to the most differentially expressed genes in tumor versus healthy control tissue (Additional file 3: Table S2) to separate samples into 3 clusters. Cluster 1 contained 91% (179/196) of total tumor samples, and 95% of samples in this cluster were tumor tissue (Fig. 4a). In contrast, tumor adjacent normal, high-risk, and healthy control samples were distributed between clusters 2 and 3. Gene set analysis demonstrated that tumor tissue also clustered separately from tumor adjacent normal and high-risk tissues based on cellular pathway alterations relative to healthy control tissue (Fig. 4b). Interestingly, the toll-like receptor (TLR) signaling pathway, best known for its microbial sensing role [36], was one of the top 10 most altered pathways in tumor tissue, with significant downregulation of *TLR4* and upregulation of *MYD88*, *IRAK1*, and other downstream genes compared to healthy control tissue (Additional file 1: Figure S3).

The NanoString gene panel further allowed us to estimate tissue immune cell abundance using cell type-specific gene signatures [37]. While similar numbers of CD45^+^ cells were present in all tissue types, total T-cells, CD8^+^ T-cells, natural killer (NK) cells, and neutrophils were enriched in tumor versus tumor adjacent normal, high-risk, and healthy control tissues (Fig. 4c). Conversely, dendritic cells and macrophages were decreased in tumor relative to non-malignant tissues. We also evaluated tissue immune infiltrates using duplex immunohistochemistry, which showed much greater densities of CD8^+^ and FOXP3^+^ cells in tumor compared to high-risk and healthy control tissues, concordant with the NanoString-based cell abundance estimates (Additional file 1: Figure S4a, b). When assessing functional immune status via a 65-plex cytokine assay, we detected significantly elevated expression of many inflammation-associated cytokines, including VEGF-A, IP-10, and IL-1RA, in tumor compared to non-tumor breast tissues (Fig. 4d).

**Fig. 5:**
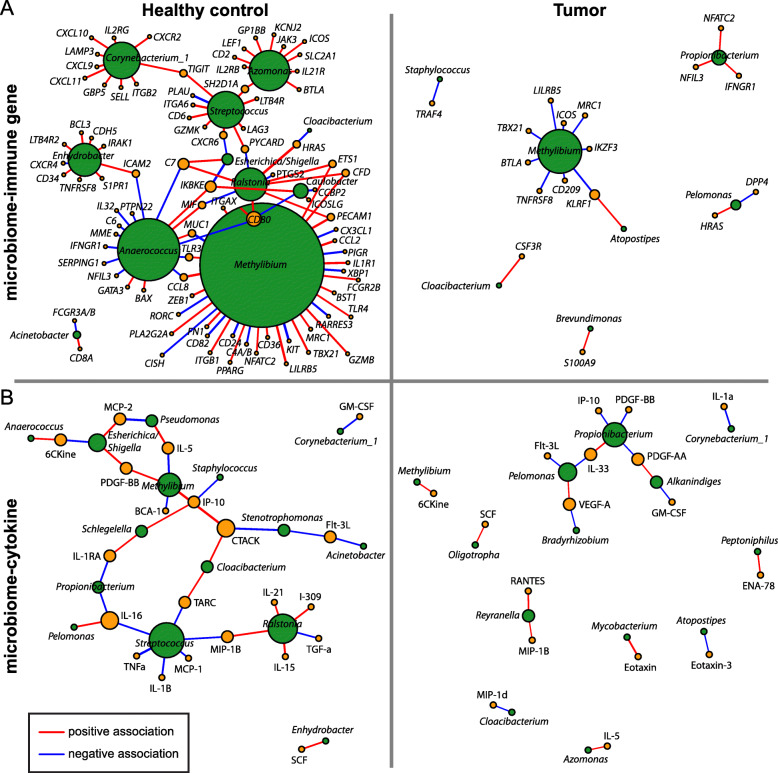
Network analyses reveal microbiome–immune associations in healthy control and tumor breast tissues. Visualization of significant microbiome associations with immune gene (**a**) and cytokine (**b**) expression based on Spearman coefficients (*p* < 0.05 for all associations shown). Each node corresponds to a single microbial (green) or immune (gold) feature, with node size proportional to the number of connections with other nodes. Edges (lines) between nodes depict positive (red) or negative (blue) associations, with edge width proportional to the magnitude of association

We then employed network analysis to identify associations between the breast microbiome and immune-related gene expression or cytokine concentrations. Our analyses focused on genes and cytokines that were differentially expressed in tumors (Fig. 4a, d). In total, 38 associations between 15 bacterial genera and 34 immune features (17 immune-related genes, 17 cytokines) were revealed in breast tumor tissue (Fig. 5). Breast tumor microbiome–immune networks consisted largely of isolated modules with bacterial nodes more likely to be connected to multiple immune nodes than vice versa; this network structure was present in tumor adjacent normal tissue as well (Additional file 1: Figure S5). In contrast, microbiome–immune networks in healthy control and high-risk breast tissues, while also organized around bacterial rather than immune nodes, contained modules that tended to be larger and more interconnected (Fig. 5, Additional file 1: Figure S5).

Within otherwise sparse tumor networks, 5 of the 15 bacterial genera had putative interactions with 2 or more immune features in the same network (Fig. 5). Of these 5 genera, 3 (*Methylibium*, *Pelomonas*, *Propionibacterium*) were identified as nodes in both microbiome–immune gene and microbiome–cytokine networks, highlighting them as top candidates for potentially influencing the intratumoral immune milieu. *Atopostipes* and *Cloacibacterium* were also present as nodes in both tumor networks, though they formed fewer connections with immune features. Meanwhile, major bacterial hubs in benign tissue networks, including *Anaerococcus*, *Caulobacter*, and *Streptococcus*, were notably missing from cancer-associated tissue networks (Fig. 5, Additional file 1: Figure S5). The genus *Methylibium*, a prominent node in healthy control networks, exhibited much lower connectivity in tumor networks.

At the pathway level, while several genes involved in TLR signaling (*TLR3*, *TLR4*, *IRAK1*) [36] co-varied positively with *Methylibium* and *Enhydrobacter* in healthy control networks, these associations were not identified in tumor networks (Fig. 5a). However, effector molecules produced downstream of TLR activation (Additional file 1: Figure S3), such as IP-10, MIP-1B, and RANTES, were significantly associated with *Propionibacterium* and *Reyranella* in tumor tissue (Fig. 5b). Multiple genes related to T-cell activation and differentiation (e.g., *CD6*, *DPP4*, *ICOS*, *IFNGR1*, *NFATC2*, *SH2D1A*, *TBX21*) [38], as well as T-cell estimated abundances, also correlated positively or negatively with bacterial genera in the breast (Fig. 5, Additional file 1: Figures S4c, S5). For instance, *Streptococcus* associated positively with *CD6*, *LAG3*, *SH2D1A*, and *TIGIT* expression and with T-cell abundance in healthy control tissue. In both healthy control and tumor tissues, *Acinetobacter* correlated positively with CD8^+^ T-cell abundance. In tumor tissue, *Methylibium* demonstrated significant negative correlations with *ICOS* and *TBX21* expression and with T-cell abundance. Finally, significant associations existed between immune features with strong ties to breast carcinogenesis and specific microbial taxa found in tumors (Fig. 5): for example, the oncogene *TRAF4* [39] co-varied negatively with *Staphylococcus*, and the proangiogenic factor VEGF-A [40] co-varied positively with *Pelomonas* and negatively with *Bradyrhizobium*. PDGF-AA and PDGF-BB, markers of poor prognosis in breast cancer [41], both co-varied negatively with *Propionibacterium*. Taken together, these results provide early evidence for potential tissue microbiome–immune interactions in breast cancer.

## Discussion

Only recently has the breast microbiome come to light, and despite data highlighting microbial differences in tissue from patients with versus without breast cancer [4–9, 35, 42], the role of tissue-resident bacteria in breast carcinogenesis remains unclear. Our results showed that multiple bacterial taxa correlate strongly with prognostic clinicopathologic features in breast cancer and that some of these taxa exhibit significant associations with immunomodulatory genes, immune cell infiltrates, and soluble factors, providing a putative basis for how microbial–immune crosstalk may influence the tumor microenvironment. Although this is the first study to identify relationships between specific breast microbial taxa and local immunity, our findings are consistent with prior work in colorectal and pancreatic cancer demonstrating that intratumoral bacteria can alter regional immune cell activation and recruitment, thereby affecting tumor development and progression [43, 44].

Numerous potential biological pathways exist through which the breast microbiota may modulate immune function and thus affect tumorigenesis. Decreased microbial diversity (Fig. 1a) or loss of keystone taxa (Fig. 2a) in breast tumors could disrupt homeostatic microbiome–immune interactions (Fig. 5), leading to immune dysregulation and carcinogenesis [2, 44]. Moreover, the low interconnectivity of tumor microbial–immune networks may reduce robustness and allow small perturbations to trigger oncogenic inflammation [2]. Connections between specific bacterial taxa and immune features may further contribute to breast cancer pathogenesis. For instance, we found that *Propionibacterium*, which includes common commensal species as well as opportunistic pathogens [45], was depleted in breast tumors (Figs. 1d and 2a); it was also positively associated with several genes related to T-cell activation and negatively associated with oncogenic growth factors (Fig. 5), suggesting that loss of this genus could promote tumor growth by downregulating adaptive antitumor responses and generating a pro-tumorigenic environment. In a similar fashion, *Staphylococcus*, another genus with reduced abundance in tumors, co-varied negatively with the expression of the known oncogene *TRAF4* (Fig. 5a). *Streptococcus* likewise demonstrated positive correlations with multiple T-cell activation genes, yet showed decreased abundance in cancer-associated samples. We postulate that deficient T-cell activation due to missing microbe-associated signals could contribute to the historically poor T-cell responses observed in breast cancer, despite the fact that we and others have noted increased lymphocyte infiltration in breast tumors compared with healthy breast tissue (Fig. 4c, Additional file 1: Figure S4b) [46, 47].

Additionally, we observed perturbations in the expression of TLR cascade members, which are canonically involved in microbial recognition (Additional file 1: Figure S3). Concurrent downregulation of several TLR genes and upregulation of downstream genes such as *MYD88* in breast tumor versus non-tumor tissues may signify negative regulatory feedback subsequent to stimulation by tumor-associated microbial products [36]. While we did not pinpoint significant associations between TLR pathway genes and specific bacteria taxa in tumors, we identified mixed relationships between various genera and TLR-induced effector molecules (Fig. 5b). Notably, a previous small study showed similar decreased expression of TLR genes in conjunction with reduced bacterial load in ER^+^ breast tumors compared to healthy control tissue [48], while prior analysis of the pancreatic tumor microbiome implicated selective TLR activation in microbiota-induced immunosuppression and oncogenesis [49], underscoring this pathway’s central role as a nexus between the microbiota, host immunity, and tumorigenesis. Further work is needed to evaluate precisely how microbe-associated immune alterations impact breast carcinogenesis and progression.

Aside from directly influencing host immune responses, the breast microbiome may produce metabolites that affect cancer and immune cells. Although an agnostic comprehensive metabolomic survey was outside the scope of this study, many bacterial genera that we identified are known to generate bioactive compounds. For example, species of the *Streptococcus* genus, which was present at much higher abundance in tissue from patients without breast cancer (Fig. 2a), synthesize cadaverine, a lysine derivative that inhibits breast tumor invasion and epithelial-to-mesenchymal transition [14, 50]. *Odoribacter*, which was present only in non-tumor tissues, includes species known to mediate antitumor activity through production of butyrate, an anti-inflammatory and tumoristatic short-chain fatty acid [14, 51]. In addition, microbiota-derived bile acids accumulate in breast tumors and correlate with decreased proliferation [52], presenting a compelling avenue for future exploration in relation to the breast microbiome.

This study is the most extensive to date using fresh-frozen surgical specimens to compare the breast microbiome in patients with versus without breast cancer [53]. For microbiome studies, particularly in low-biomass tissues, fresh-frozen samples are superior to formalin-fixed paraffin-embedded (FFPE) samples because the latter suffer from DNA degradation and greater risk of microbial contamination during formalin fixation and archival storage [13, 54]. Interestingly, while studies using fresh-frozen breast samples have found reduced or similar bacterial α-diversity in tumor versus non-tumor tissues (Fig. 1a) [4, 8, 9, 55], recent work based on FFPE samples showed the opposite trend [13]. Our large sample size provided sufficient power to examine microbial associations with multiple covariates, including histologic subtype and node-positive status (never previously investigated), and detect taxa-level differences in relative abundances even after correcting for multiple comparisons. Unlike previous studies, we performed the additional step of adjusting our comparisons for known confounders (e.g., age, race) that were significantly different between patients with and without cancer, decreasing the false positive rate. Due to the challenges of working with low-biomass tissue such as the breast [15], we implemented numerous other measures to exclude false positives (contaminants), including (1) working with UV-irradiated pipettes and disposables in a dedicated laminar flow hood and (2) procuring operating room controls from each institution from which we requested tissue and computationally removing ASVs found in these controls, or in DNA extraction and library preparation negative controls, from all samples (Additional file 1: Figure S1).

Consistent with previous reports [13, 42, 55], we found that the dominant bacterial phylum in breast tissues was Proteobacteria, followed by Firmicutes and Actinobacteria (Fig. 1b). We also measured lower abundance of *Enterobacteriaceae* and higher abundances of *Corynebacterium*, *Lactococcus*, and *Streptococcus* in breast tissue from healthy patients compared to those with cancer (Figs. 1c and 2a), in accordance with prior work [5]. However, the same study describes a positive association between *Staphylococcus* and adjacent normal versus healthy control tissue [5], whereas we note the opposite relationship. Another group observed that the abundance of *Alkanindiges* correlated with ER^−^ tumors [13], which agrees with our findings (Fig. 3a). Although we identified other bacterial profiles corresponding with breast tumor receptor status (Fig. 3a–d), these profiles were distinct from though not contradictory to those described previously [4, 7, 9, 13, 35]. Overall, it remains difficult to directly compare breast microbiome data from individual studies for myriad reasons, including differences in tissue source (fresh-frozen vs FFPE, surgery vs biopsy, geography), experimental protocols (tissue collection, DNA extraction, library preparation, sequencing), bioinformatic pipelines, and statistical methods that influence results [33]. Ideally, a standardized protocol for breast microbiome studies should be developed and universally adopted. Future work should also investigate associations between the breast microbiome and other clinical factors known to correlate with breast cancer risk, including patient body mass index and race [9], to determine whether these factors may additionally influence breast microbial composition.

Like other cross-sectional patient-based microbiome studies, our findings are limited by the inability to determine causality. For instance, the negative relationship between *Propionibacterium* and IP-10 (Fig. 5b) could indicate that the microbe inhibits cytokine production, the cytokine hinders microbe growth, or both. We intended instead to provide detailed characterization of breast microbial and immune signatures that co-varied and correlated with clinicopathologic data as a springboard for future preclinical and clinical work. Even if certain microbial patterns result from, rather than drive, pro- or anti-tumorigenic immune alterations, this knowledge could potentially be applied as a biomarker for breast cancer susceptibility or prognosis. In this context, our identification of subtle microbial differences between histologically normal breast tissues from patients at normal versus high risk for breast cancer (Fig. 2a) merits replication in larger studies. The high-risk patient group, consisting of individuals with well-documented breast cancer risk factors who were nonetheless histologically cancer-free at the time of the study, represents particularly fertile ground for future investigation: correlating differences between healthy control and high-risk tissues with longitudinal outcomes (e.g., cancer development) could increase our understanding of factors associated with cancer initiation and facilitate the development of better approaches for cancer detection and prevention.

Our results also encourage further examination of routes through which microbes may colonize the breast tissue, including translocation from the gut and passage via the nipple. Specifically, dendritic cells have been shown to carry live commensal bacteria and may facilitate bacterial transport from the gut lumen to the breast, especially during pregnancy and lactation [56, 57]. Further supporting the existence of a gut–breast axis are observations that probiotic *Lactobacillus* strains can be found in the mammary gland after oral ingestion [58] and that dietary changes can influence breast microbiome profiles [59]. Meanwhile, skin and oral bacteria may travel through nipple–areolar orifices to populate the breast tissue, with the latter contacting the nipple during breastfeeding or sexual activity [6]. Although some compositional overlap is present, previous work indicates that the breast microbiome is distinct from that of the gut, oral cavity, and overlying skin, suggesting that environmental factors such as pH, available nutrients, and oxygen levels may select for certain dominant taxa following initial colonization [4, 6, 42, 53]. Additional preclinical and clinical studies that concurrently characterize the microbiome at multiple sites in the same individuals are essential for clarifying these potential bacterial entry routes and may pave the way for breast microbial manipulation as a potential therapeutic modality or adjunct.

## Conclusions

In conclusion, we provide evidence supporting a novel role for local microbiome–immune crosstalk in breast cancer and delineate breast microbial profiles associated with multiple prognostic clinical variables. This work sets the stage for further studies assessing causative mechanisms whereby microbial–immune interactions influence breast cancer development and progression. As our understanding of the breast microbiome increases, it may become possible to use diet [59], probiotics [60], selective antibiotics [49], or fecal microbiota transplant [2, 61] as well as topical, injected, or surgically applied agents to establish a more anti-tumorigenic breast microbiome to treat or, better yet, prevent breast cancer.

## Supplementary Information

**Additional file 1: **Five supporting figures, with corresponding figure captions provided within the file. **Figure S1.** R code for removing ASVs detected in environmental/negative controls, and bacterial and non-eukaryotic ASVs characterized in environmental/negative controls. **Figure S2.** Total bacterial load is significantly higher in tissues versus environmental/negative controls and is similar across tissue types. **Figure S3.** Breast tumor tissue exhibits upregulation of multiple downstream toll-like receptor pathway genes. **Figure S4.** T-cell infiltration and association with specific bacterial genera vary by breast tissue type. **Figure S5.** Network analyses reveal microbiome–immune associations in high-risk and tumor adjacent normal breast tissues.

**Additional file 2: Table S1.** Differentially abundant bacterial taxa according to clinicopathologic features (tissue type, TNM stage, histologic grade, histology).

**Additional file 3: Table S2.** Differentially expressed genes between tumor (*n* = 196) versus healthy control (*n* = 55) breast tissue. Inclusion criteria were |fold change| > 2 and FDR <  0.05.

## Data Availability

The datasets used and/or analyzed during the current study are included in this published article, additional supporting files, and in the European Nucleotide Archive (ENA), study accession number PRJEB43655 [62].

## Abbreviations

ASV: Amplicon sequence variant
DADA: Divisive Amplicon Denoising Algorithm
ER: Estrogen receptor
FFPE: Formalin-fixed paraffin-embedded
HER2: Human epidermal growth factor 2
HRT: Hormone replacement therapy
IDC: Invasive ductal carcinoma
ILC: Invasive lobular carcinoma
LVI: Lymphovascular invasion
NK: Natural killer
OCP: Oral contraceptive pill
OTU: Operating taxonomic unit
PR: Progesterone receptor
TLR: Toll-like receptor
TNBC: Triple-negative breast cancer
