# Supplementary material for: Human breast microbiome correlates with prognostic features and immunological signatures in breast cancer
Source: Genome Med. 2021 Apr 16;13:60. doi: 10.1186/s13073-021-00874-2 (PMC8052771; doi:10.1186/s13073-021-00874-2)
Supplement: Supplementary file 1 — Additional file 1: Five supporting figures, with corresponding figure captions provided within the file. Figure S1. R code for removing ASVs detected in environmental/negative controls, and bacterial and non-eukaryotic ASVs characterized in environmental/negative controls. Figure S2. Total bacterial load is significantly higher in tissues versus environmental/negative controls and is similar across tissue types. Figure S3. Breast tumor tissue exhibits upregulation of multiple downstream toll-like receptor pathway genes. Figure S4. T-cell infiltration and association with specific bacterial genera vary by breast tissue type. Figure S5. Network analyses reveal microbiome–immune associations in high-risk and tumor adjacent normal breast tissues. [file 13073_2021_874_MOESM1_ESM.docx]

# Supplementary figures

**R code for removing ASVs detected in environmental/negative controls:**

prune_negatives = function(physeq, negs, samps) {

negs.n1 = prune_taxa(taxa_sums(negs)>=1, negs)

samps.n1 = prune_taxa(taxa_sums(samps)>=1, samps)

allTaxa <- names(sort(taxa_sums(physeq),TRUE))

negtaxa <- names(sort(taxa_sums(negs.n1),TRUE))

taxa.noneg <- allTaxa[!(allTaxa %in% negtaxa)]

return(prune_taxa(taxa.noneg,samps.n1))

}

**Bacterial and non-eukaryotic ASVs characterized in environmental/negative controls (and subsequently removed from all samples):**

|  | **Domain_Phylum_Class_Order_Family_Genus_Species** | | | | | |
| --- | --- | --- | --- | --- | --- | --- |
| ASV7265 | Bacteria_Actinobacteria_Actinobacteria_Micrococcales_Dermacoccaceae_NA_NA | | | | |  |
| ASV8533 | Bacteria_Actinobacteria_Actinobacteria_Micrococcales_Micrococcaceae_NA_NA | | | | |  |
| ASV3871 | Bacteria_Actinobacteria_Actinobacteria_NA_NA_NA_NA | | |  |  |  |
| ASV8056 | Bacteria_Acidobacteria_Solibacteres_Solibacterales_Solibacteraceae_(Subgroup_3)_Bryobacter_NA | | | | | |
| ASV1023 | Bacteria_Acidobacteria_Solibacteres_Solibacterales_Solibacteraceae_(Subgroup_3)_Bryobacter_NA | | | | | |
| ASV21023 | NA_NA_NA_NA_NA_NA_NA |  |  |  |  |  |
| ASV21025 | NA_NA_NA_NA_NA_NA_NA |  |  |  |  |  |
| ASV21026 | NA_NA_NA_NA_NA_NA_NA |  |  |  |  |  |

**Additional file 1: Figure S1.** R code for removing ASVs detected in environmental/negative controls, and bacterial and non-eukaryotic ASVs characterized in environmental/negative controls.

**
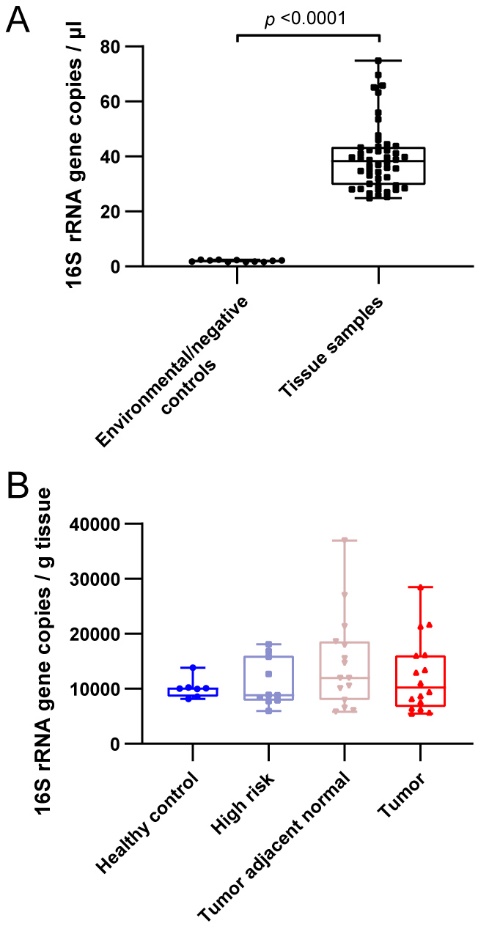
**

**Additional file 1: Figure S2.** Total bacterial load is significantly higher in tissues versus environmental/negative controls and is similar across tissue types. 16S rRNA gene copy numbers in controls and breast tissue samples quantified by qPCR with universal bacterial primers and normalized by volume (**a**) or tissue mass (**b**). Box plots show median and interquartile range. *P-*value results from Mann-Whitney test; *n* = 11 environmental/negative controls and 7 healthy control, 10 high-risk, 15 tumor adjacent normal, 16 tumor samples.


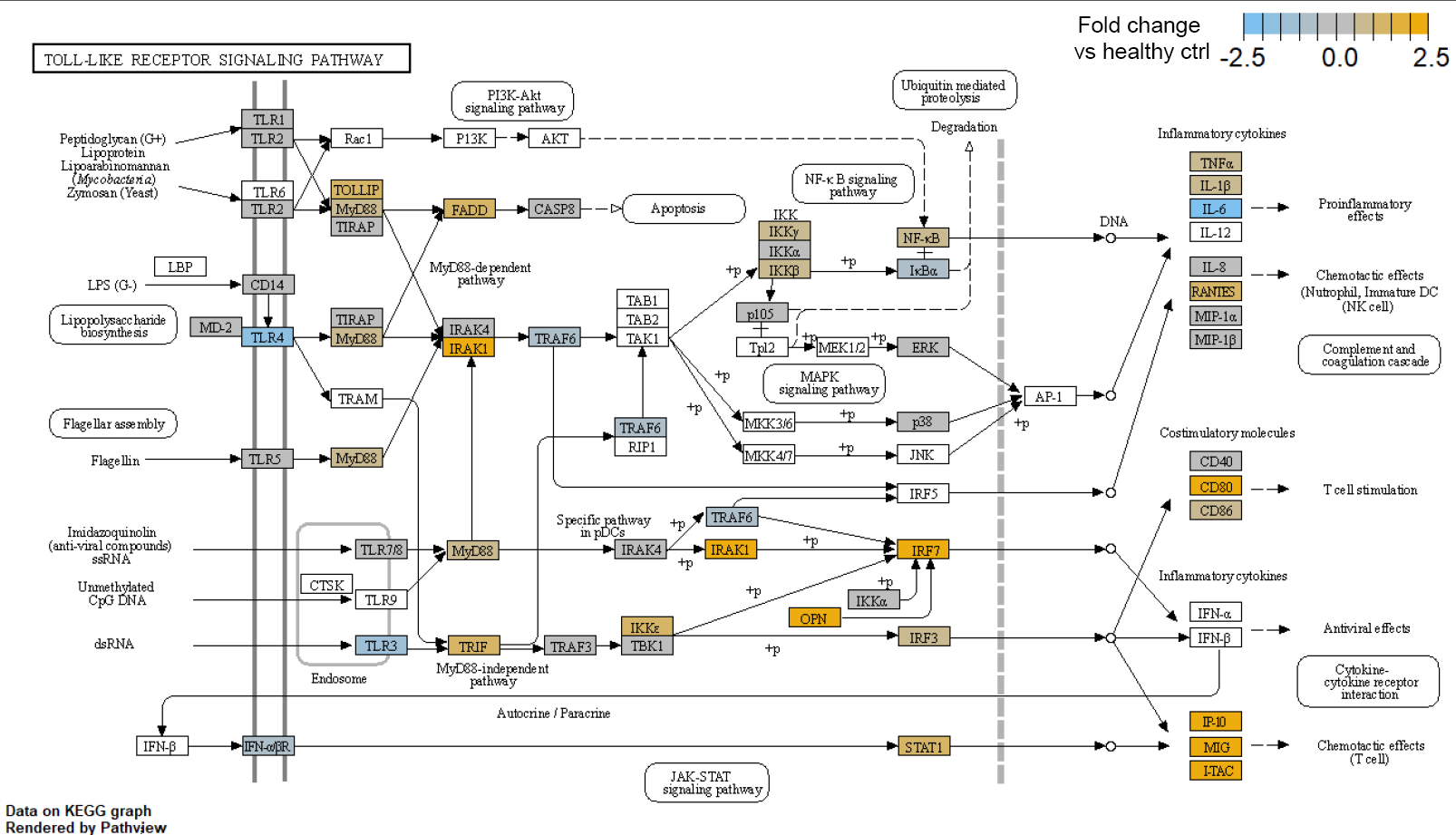

**Additional file 1: Figure S3.** Breast tumor tissue exhibits upregulation of multiple downstream toll-like receptor pathway genes. Differential gene expression in breast tumor versus healthy control tissue based on NanoString data overlaid on the KEGG toll-like receptor signaling pathway. Colored boxes indicate genes that were significantly upregulated (gold) or downregulated (blue) in tumor tissue at a threshold of *p* < 0.05 by linear regression analysis.


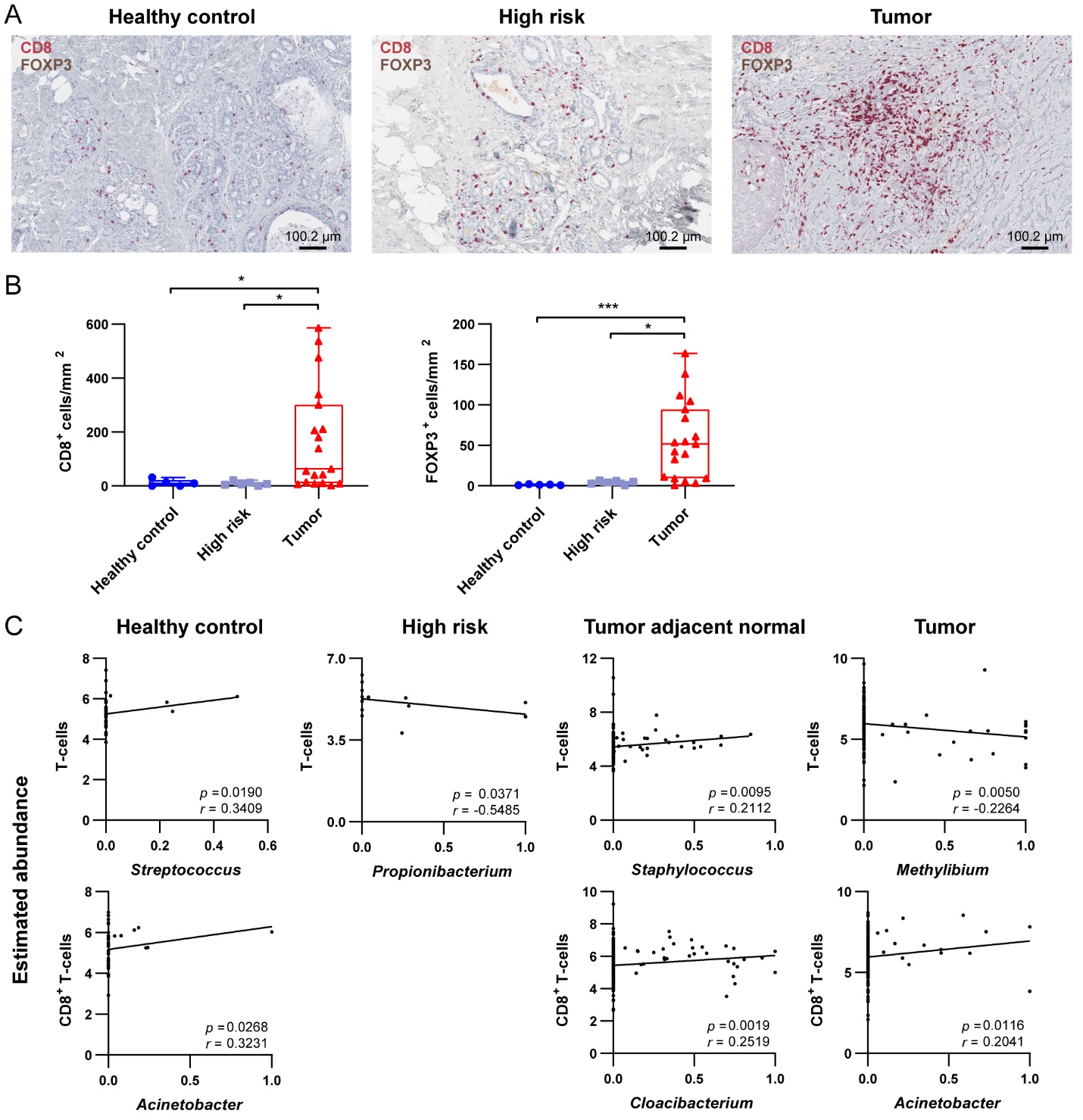


**Additional file 1: Figure S4.** T-cell infiltration and association with specific bacterial genera vary by breast tissue type. Immunohistochemical staining for CD8 (red) and FOXP3 (brown) performed on a representative subset of specimens (**a**) and quantified per unit area (**b**). Box plots show median and interquartile range. **p* < 0.05, ****p* < 0.001 by Kruskal-Wallis test with posthoc Dunn test; *n* = 5 healthy control, 6 high-risk, 19 tumor samples. (**c**) Significant Spearman correlations between T-cell and CD8^+^ T-cell estimated abundances (based on NanoString data) and bacterial genera according to breast tissue type.


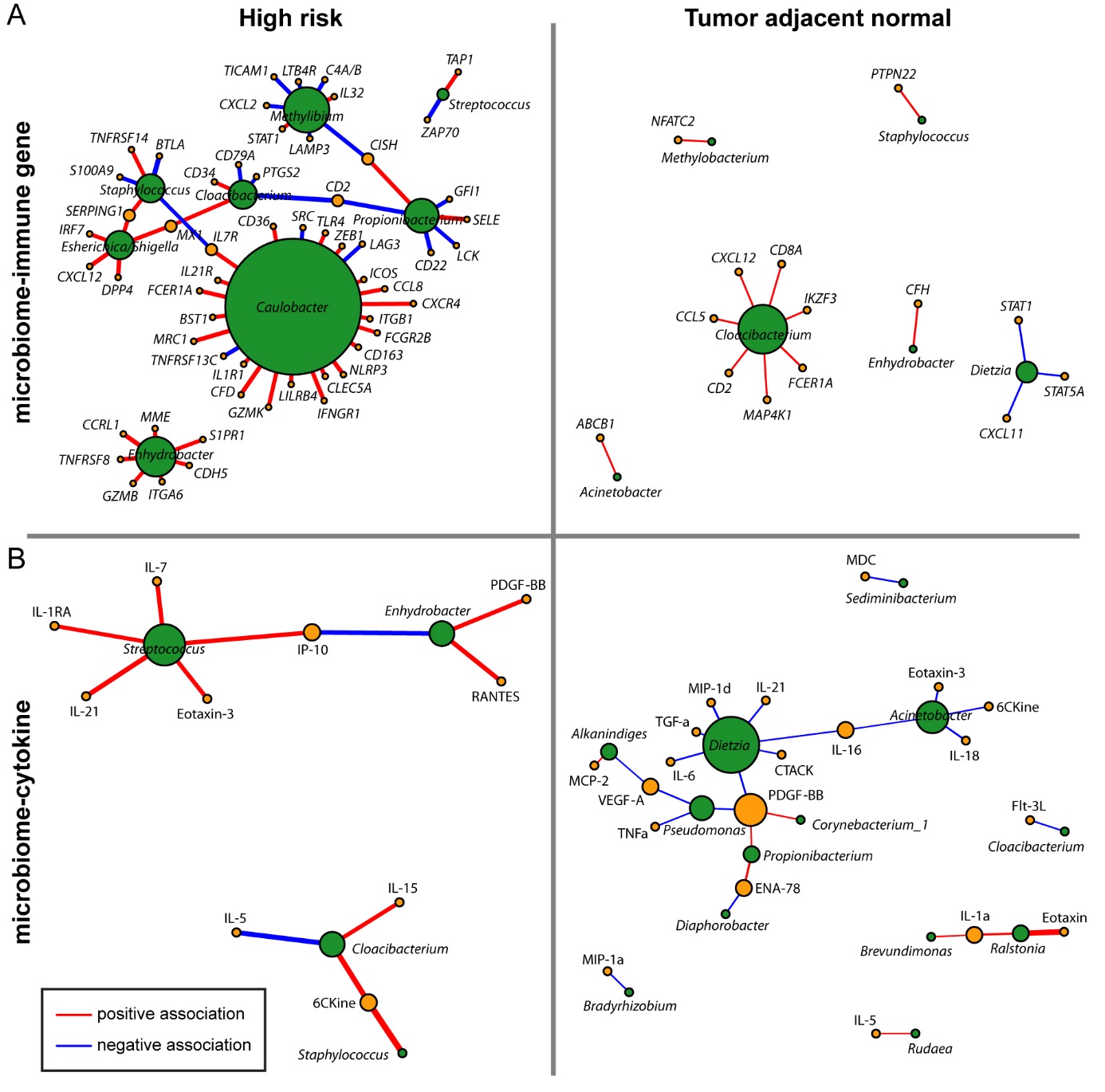


**Additional file 1: Figure S5.** Network analyses reveal microbiome–immune associations in high-risk and tumor adjacent normal breast tissues. Visualization of significant microbiome associations with immune gene (**a**) and cytokine (**b**) expression based on Spearman coefficients (*p* < 0.05 for all associations shown). Each node corresponds to a single microbial (green) or immune (gold) feature, with node size proportional to the number of connections with other nodes. Edges (lines) between nodes depict positive (red) or negative (blue) associations, with edge width proportional to the magnitude of association.
